# The influence of stress perception on mobile phone addiction tendency in nursing undergraduates: the mediating role of self-control and the moderating role of psychological capital

**DOI:** 10.1186/s12912-025-03753-y

**Published:** 2025-08-25

**Authors:** Xiangxiang Li, Meifang Wang, Xiujuan Feng, Lang He, Juan Du

**Affiliations:** 1 Department of Nursing and Rehabilitation, Xi ’an Jiaotong University City College, Xi’an, 710018 China; 2 School of Nursing, The Fourth Military Medical University, Xi’an, 710032 China

**Keywords:** Nursing undergraduates, Stress perception, Self control, Psychological capital, Mobile phone addiction

## Abstract

**Background:**

Nursing students face significant pressure due to academic burdens and clinical demands, which may increase their risk of mobile phone addiction. Mobile phone addiction impairs students’ psychosocial functioning and can lead to depression, anxiety, loneliness, inattention, and other adverse mental and physical health outcomes. This study addresses a critical gap in the literature by examining how stress perception, self-control, and psychological capital interact to influence mobile phone addiction among nursing undergraduates, a population uniquely exposed to high academic and emotional stressors. Understanding these factors is essential for designing effective interventions to reduce mobile phone addiction in this vulnerable group.

**Methods:**

From September 2022 to November 2022, a stratified random sampling method was used to select 616 nursing undergraduates from a university in Shaanxi Province as the investigation objects. Subjective stress, self-control ability, mobile phone addiction tendency and psychological capital level of nursing undergraduates were evaluated by pressure perception scale, self-control scale, mobile phone addiction tendency and positive psychological capital questionnaire. The mediating effect was tested by percentile Bootstrap method with deviation correction, and the moderating effect was tested by PROCESS program.

**Results:**

(1) Among the 592 valid questionnaires collected, 176 (29.72%) nursing undergraduates exhibited mobile phone addiction; (2) Self-control partially mediated the relationship between stress perception and mobile phone addiction tendency, with an effect value of 0.229, accounting for 59.02% of the total effect; (3) Psychological capital moderated the relationship between self-control and mobile phone addiction tendency, with the influence of self-control increasing as psychological capital levels rose (*β*= -0.099, *t* = -4.255, *p* < 0.001).

**Conclusions:**

This study highlights the novel role of psychological capital as a moderator in the relationship between self-control and mobile phone addiction among nursing undergraduates. The findings suggest that interventions aimed at enhancing self-control and fostering psychological capital could effectively reduce mobile phone addiction in this population. These results provide practical implications for nursing educators and mental health professionals, emphasizing the need for targeted stress management and resilience-building programs to support nursing students’ well-being.

**Supplementary Information:**

The online version contains supplementary material available at 10.1186/s12912-025-03753-y.

## Introduction

Mobile phone addiction tendency refers to a new type of behavioral addiction in which individuals frequently and excessively use mobile phones and cannot control such use behavior through autonomous consciousness, which not only causes damage to their social function but also leads to adverse psychological and behavioral problems [1]. Mobile phone addiction tendency impairs an individual’s daily psychosocial functioning, and in severe cases, it can lead to depression, anxiety, loneliness, lack of attention, cognitive impairment, decreased immunity, decreased vision, and sleep disorders [2–4]. Epidemiological investigations have shown that the global prevalence of mobile phone addiction tendency is 26.99% [5]. Compared with other adults, the incidence of mobile phone addiction tendency in college students is higher, with the prevalence among traditional Chinese medicine students reaching 41.93% [6], and this proportion is still growing rapidly. Medical students, including nursing undergraduates, face greater academic pressure than other students, and studies have shown that high academic pressure is related to mobile phone addiction tendency [7]. Nursing undergraduates are an important reserve force for clinical nursing work. After graduation, they will become nurses who shoulder the responsibility of saving lives. The mental health of healthcare service personnel has important implications for the quality of medical work and the relationship between nurses and patients. Therefore, it is crucial to focus on the mental health status of nursing students and clarify the factors that influence mobile phone addiction.

This study is guided by the General Strain Theory (GST) [8], which posits that stress leads to negative emotions and maladaptive behaviors, such as addiction, as a coping mechanism. Building on this framework, we also incorporate the Strength Model of Self-Control [9] and Psychological Capital Theory [10] to explore how self-control and psychological capital mediate and moderate the relationship between stress perception and mobile phone addiction among nursing undergraduates. These theoretical frameworks provide a robust foundation for understanding the mechanisms underlying mobile phone addiction in this population.

Studies have found that the pressure caused by the academic burden of medical students at the university stage may induce a series of behaviors that are not conducive to the healthy development of students’ physical and mental health, such as anxiety, depression, suicidal ideation, procrastination behavior, and mobile phone addiction tendency [11, 12]. However, the internal mechanisms of how stress perception affects mobile phone addiction tendency remain unclear. Therefore, the purpose of this study is to explore the direct and indirect effects of stress perception on mobile phone addiction, with a focus on the mediating role of self-control and the moderating role of psychological capital, and to provide new ideas for designing strategies to improve mobile phone addiction tendency in nursing undergraduates.

### Stress perception and mobile phone addiction

Stress perception refers to an individual’s assessment of whether a stimulus event creates pressure on themselves [13]. High perceived stress is positively correlated with anxiety, depression, and addictive behavior [14]. Recent meta-analyses and studies have further validated these relationships, demonstrating that stress perception is a robust predictor of mental health issues and maladaptive behaviors, including mobile phone addiction. For example, a 2021 meta-analysis by Liu et al. found that perceived stress significantly predicts addictive behaviors, with mobile phone addiction being one of the most prevalent outcomes among college students [15]. Similarly, a 2022 study by Zhang et al. highlighted that stress perception mediates the relationship between academic pressure and mobile phone addiction, particularly in high-stress populations such as medical and nursing students [14].

For nursing undergraduates, they face many pressures during their campus period, such as completing their studies, adapting to the environment, interpersonal communication, dating and making friends, employment, and transitioning from nursing students to clinical nurses. The interweaving of multiple tasks causes students to experience a significant pressure load. According to the General Strain Theory (GST) [8], various behavior problems arise from negative experiences caused by stress or tension and are seen as measures to reduce perceived stress. Recent research has expanded on GST by showing that stress-induced behaviors, such as mobile phone addiction, are particularly prevalent in populations with high academic and emotional demands, such as nursing students [16]. In other words, mobile phone addiction tendency is a way for college students to release pressure or tension. Therefore, this study proposes Hypothesis 1: Stress perception positively predicts mobile phone addiction among nursing undergraduates.

### The mediating role of Self-Control

Self-control is an individual’s internal ability to resist external temptation and achieve goals. Seminal works by Tangney et al. (2004) define self-control as a critical factor in regulating behavior and achieving long-term goals, particularly in high-stress environments [17]. Some studies have found that stress perception has a significant negative predictive effect on self-control [18]. According to the Strength Model of Self-Control [9], energy resources can positively affect self-control levels, and any activity that consumes energy resources, such as making choices and emotional control, will lead to the decline or failure of self-control levels. Baumeister et al. (2007) further emphasize that self-control operates like a muscle, which can be depleted under stress but strengthened through regular practice [9]. Stress loads deplete individual energy resources and weaken self-control. Previous studies have confirmed that self-control significantly negatively predicts mobile phone addiction tendency. According to the Use-Satisfaction Theory [19], as a universal medium for information acquisition and communication, individuals can obtain satisfaction and happy experiences from mobile phones. If individuals over-rely on this psychological experience and do not control their own behavior, they may eventually develop mobile phone addiction tendency [20]. Therefore, this study proposes Hypothesis 2: Self-control mediates the relationship between stress perception and mobile phone addiction tendency among nursing undergraduates.

### The moderating role of psychological capital

Psychological capital is defined as a positive psychological state shown by an individual in the process of growth and development [10]. Luthans et al. (2007) conceptualize psychological capital as comprising four key components: self-efficacy, hope, resilience, and optimism, which collectively enhance an individual’s ability to cope with stress and adversity [21]. Baumeister et al. [9] believe that self-control needs to consume psychological resources. In a certain period, the success of self-control depends on the amount of internal psychological resources an individual possesses. Psychological capital is a positive psychological resource owned by an individual, which can be used as a supplement to the consumption of self-control ability. The higher the level of psychological capital, the more psychological resources an individual can mobilize within the body, and the stronger the self-control ability, which is consistent with Dong Yaqi et al. [22]. Recent studies have shown that psychological capital acts as a buffer against stress, enabling individuals to maintain self-control even under high-pressure conditions [14].

In this study, psychological capital as a moderating variable is supported by the Ego Depletion Theory [23], which posits that psychological resources are limited, and when facing stressors, individuals will mobilize internal resources to resist. If the consumption of internal resources is not timely supplemented, the mobilization will be blocked after exhaustion, leading to serious mental health problems. Individuals with high psychological capital have a more flexible cognitive behavior model and are more likely to obtain energy from the outside world when coping with adverse stress [24]. Specifically, psychological capital enhances self-control by providing individuals with the resilience to recover from stress and the optimism to maintain motivation, thereby reducing the likelihood of engaging in maladaptive behaviors such as mobile phone addiction [17]. Studies have found that the higher the level of psychological capital, the stronger the self-control ability, and when facing stressors, individuals are more likely to conform their behavior to public expectations and reduce pressure relief through problem behaviors, such as addiction to the Internet or games. Therefore, this study proposes Hypothesis 3: Psychological capital moderates the relationship between self-control and mobile phone addiction tendency among nursing undergraduates.

**Fig. 1:**
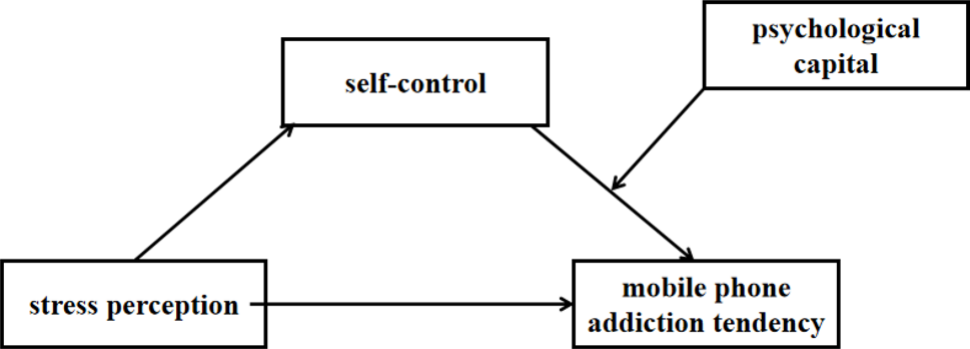
The hypothetical study model

Based on the above analysis and theoretical frameworks, this study aims to explore the relationship between mobile phone addiction tendency and stress perception, self-control, and psychological capital among nursing undergraduates. The findings will contribute to the literature by providing a deeper understanding of the mechanisms underlying mobile phone addiction in this population and offering practical implications for intervention strategies (Fig. 1).

## Methods

### Research object

From September 2022 to November 2022, undergraduate nursing students in a university in Shaanxi Province were selected as the survey objects. Stratified random sampling was conducted by grade (freshman, sophomore, and junior, senior) to ensure representativeness across all academic years. The inclusion criteria were: (1) four-year nursing undergraduates; (2) Nursing students who voluntarily sign informed consent. Exclusion criteria: (1) Those not in school due to reasons such as suspension or military service.

The sample size was calculated using the formula N = Z² × [P × (1-P)] / E², where N is the sample size, Z is the statistic (1.96 for 95% confidence), E is the margin of error (4%), and P is the estimated prevalence of mobile phone addiction among medical students (42%, based on previous studies [6]). Considering a 5% non-response rate, the required sample size was 616 participants.

### Survey tools

#### Chinese version of the stress perception scale

This scale, adapted by Professor Yang Yanzhong from the English version of the Perceived Stress Scale (PSS) [13, 25], and consists of 14 items across two dimensions: feeling out of control (7 items) and feeling tense (7 items). A 5-point Likert scale was used, with scores ranging from 1 (“never”) to 5 (“always”). Reverse-scored items were adjusted accordingly. The total score ranges from 14 to 70, with higher scores indicating higher stress perception. The scale has demonstrated good reliability and validity in previous studies (Cronbach’s *α* = 0.882) [26]. In this study, Cronbach’s *α* was 0.741.

#### Self-control scale

Adapted from the Self-Control Scale (SCS) by Tangney et al. [27] and modified by Tan Shuhua et al. for the Chinese context, this scale includes 19 items across five dimensions: impulse control (6 items), healthy habits (3 items), resisting temptation (4 items), focusing on work (3 items), and limiting entertainment (3 items). A 5-point Likert scale was used, with scores ranging from 1 (“completely inconsistent”) to 5 (“completely consistent”). Reverse-scored items were adjusted accordingly. The total score ranges from 19 to 95, with higher scores indicating greater self-control. The scale has demonstrated good reliability and validity in previous studies (Cronbach’s *α* = 0.853) [28]. In this study, Cronbach’s *α* was 0.879.

#### Positive psychological capital questionnaire

Developed by Zhang Kuo et al. [29], this questionnaire includes 26 items across four dimensions: self-efficacy (7 items), hope (6 items), optimism (6 items), and resilience (7 items). A 7-point Likert scale was used, with scores ranging from 1 (“completely inconsistent”) to 7 (“completely consistent”). The total score ranges from 26 to 182, with higher scores indicating higher psychological capital. The scale has demonstrated good reliability and validity in previous studies (Cronbach’s *α* = 0.918) [30]. In this study, Cronbach’s *α* was 0.928.

#### College students’ mobile phone addiction tendency scale

Developed by Xiong Jie et al. [31], this scale includes 16 items across four dimensions: withdrawal symptoms (6 items), highlighting behaviors (4 items), social soothing (3 items), and mood changes (3 items). A 5-point Likert scale was used, with scores ranging from 1 (“very inconsistent”) to 5 (“very consistent”). The total score ranges from 16 to 80, with scores ≥ 48 indicating mobile phone addiction. The scale has demonstrated good reliability and validity in previous studies (Cronbach’s *α* = 0.895) [32]. In this study, Cronbach’s *α* was 0.927.

An additional survey questionnaire file shows this in more detail [see Additional file 1].

### Survey method

#### Stratified random sampling was implemented as follows

1. The ratio of the sample size to the population was determined (616:1124 ≈ 6:11).
2. The sample ratio was used to determine the number of participants to be selected from each grade: 62 freshmen, 55 sophomores, 273 juniors, and 226 seniors.
3. Simple random sampling was conducted within each grade. For example, in a given grade, all eligible students were numbered, and numbers were drawn from a non-transparent container without replacement until the required number of participants (m) was reached.

#### To minimize response bias, the following measures were taken

1. Participants were assured of anonymity to encourage honest responses.
2. Reverse-scored items were included to reduce acquiescence bias.
3. Data collection was conducted during regular class hours to ensure a controlled environment.

In total, 616 questionnaires were distributed, and 592 valid responses were collected, yielding an effective response rate of 96.10%.An additional original data file shows this in more detail [see Additional file 2].

### Data processing

Data were analyzed using SPSS 27.0. Descriptive statistics, including means and standard deviations, were calculated for all variables. T-tests or ANOVA were used to compare scores across demographic groups. Pearson correlation analysis was conducted to explore relationships between variables.

For mediation and moderation analyses, the PROCESS macro for SPSS (Model 4 and Model 14) was used. Mediation analysis tested whether self-control mediated the relationship between stress perception and mobile phone addiction tendency. Moderation analysis examined whether psychological capital moderated the relationship between self-control and mobile phone addiction tendency. The bias-corrected percentile Bootstrap method (5,000 resamples) was used to test the significance of indirect effects, with 95% confidence intervals.

To address potential common method bias, Harman’s single-factor test was conducted. All items from the four scales were subjected to exploratory factor analysis, which yielded 11 factors with eigenvalues greater than 1. The variance explained by the first factor was 21.98%, below the 40% threshold, indicating that common method bias was not a significant concern in this study.

## Results

### Comparison of scores of nursing undergraduates with different demographic characteristics on various scales

There were 176 participants with mobile phone addiction (29.72%), and other specific information is shown in Table 1.

**Table 1:** Comparison of scores of nursing undergraduates with different demographic characteristics in each scale

Variable		*n*	PSS	SCS	PPQ	MPATS
Sex	male	34	39.79 ± 6.71	63.74 ± 10.02	101.65 ± 19.64	40.59 ± 9.34
	female	558	39.75 ± 6.25	62.35 ± 10.35	101.33 ± 16.64	41.02 ± 9.98
	*t*		0.036	0.760	0.108	-0.245
	*p*		0.977	0.447	0.914	0.807
Grade	Freshman	61	38.13 ± 6.96	61.70 ± 10.44	102.84 ± 17.34	40.38 ± 9.79
	Sophomore	54	40.30 ± 5.40	62.72 ± 8.06	99.63 ± 14.92	39.70 ± 10.51
	Junior	268	40.05 ± 6.34	62.27 ± 10.71	101.22 ± 17.31	42.31 ± 10.12
	Senior	209	39.72 ± 6.16	62.77 ± 10.37	101.51 ± 16.53	39.81 ± 9.44
	F		1.701	0.209	0.357	2.968
	*p*		0.166	0.890	0.784	0.031

Note: PSS, Stress Perception Scale; SCS, Self-control Scale; PPQ, Positive Psychological Capital Questionnaire; MPATS, Mobile Phone Addiction Tendency Scale for College students

### Correlation between stress perception, self-control, positive psychological capital and mobile phone addiction tendency

As presented in Table 2, PPS score of nursing undergraduates (39.76 ± 6.27) was negatively correlated with SCS score (62.43 ± 10.33) and PPQ score (101.34 ± 16.81) (*r* = -0.576, -0.673, both *p* < 0.001). It was positively correlated with MPATS score (40.99 ± 9.94) (*r* = 0.386, *p* < 0.001). SCS scores were positively correlated with PPQ scores (*r* = 0.572, *p* < 0.001), and negatively correlated with MPATS scores (*r*= -0.487, *p* < 0.001). PPQ score was negatively correlated with MPATS score (*r* =-0.338, *p* < 0.001).

**Table 2:** Bivariate correlation analysis

	M	SD	1	2	3	4
1. PSS	39.76	6.27	1			
2. SCS	62.43	10.33	-0.576***	1		
3. PPQ	101.34	16.81	-0.673***	0.572***	1	
4. MPATS	40.99	9.94	0.386***	-0.487***	-0.338***	1

Note: PSS, Stress Perception Scale; SCS, Self-control Scale; PPQ, Positive Psychological Capital Questionnaire; MPATS, Mobile Phone Addiction Tendency Scale for College students

* *p* < 0.05, ** *p* < 0.01,*** *p* < 0.001

### Mediated effect test with adjustment

According to the views of Wen Zhonglin et al. [33], the mediating role of self-control in stress perception and mobile phone addiction tendency was first examined, followed by the moderating role of positive psychological capital.

Firstly, regression analysis was conducted with PSS score as independent variable and MPATS score as dependent variable (controlling gender and grade). The results showed that PSS score had significant positive predictive effect on MPATS score (*β* = 0.388, SE = 0.060, *p* < 0.001). The overall effect of PSS score on MPATS score was significant. Then the PROCESS program of SPSS 27.0 (Model 4) was used to test the mediation effect. The sampling times of Bootstrap test was 5000, and gender and grade were controlled. The results showed that PSS score negatively predicted SCS score (*β* = -0.579, SE = 0.033, *p* < 0.001), when both PSS score and SCS score predicted MPATS score simultaneously, PSS score (*β* = 0.159, SE = 0.044, *p* < 0.001) and SCS scores (*β* = -0.395, SE = 0.044, *p* < 0.001) had significant predictive effect. The corrected Bootstrap test showed that the mediating effect of self-control was significant, the indirect effect value was 0.229, the 95% confidence interval was [0.165, 0.296], and the mediating effect accounted for 59.02% of the total effect (0.388).

Model 14 of PROCESS was used to test the mediated effect. Taking gender and grade as control variables, the results showed that SCS score ×PPQ score negatively predicted MPATS score (Table 3), suggesting that psychological capital plays a moderating role between self-control and mobile phone addiction tendency.

**Table 3:** Test the moderating effect of positive psychological capital

Dependent Variable		β	SE	t	95% CI	*R* ^2^	F
SCS						0.336	99.216***
	PSS	-0.579	0.034	-17.207***	[-0.453~-0.706]		
MPATS						0.277	37.289***
	PSS	0.179	0.051	3.531***	[0.079 ~ 0.278]		
	SCS	-0.371	0.046	-8.143***	[-0.461~-0.282]		
	PPQ	0.010	0.050	0.201	[-0.088 ~ 0.109]		
	SCS×PPQ	-0.099	0.023	-4.255***	[-0.144~-0.053]		

Note: PSS, Stress Perception Scale; SCS, Self-control Scale; PPQ, Positive Psychological Capital Questionnaire; MPATS, Mobile Phone Addiction Tendency Scale for College students

* *p* < 0.05, ** *p* < 0.01,***p < 0.001

All variables are standardized and then brought into the regression equation

**Fig. 2:**
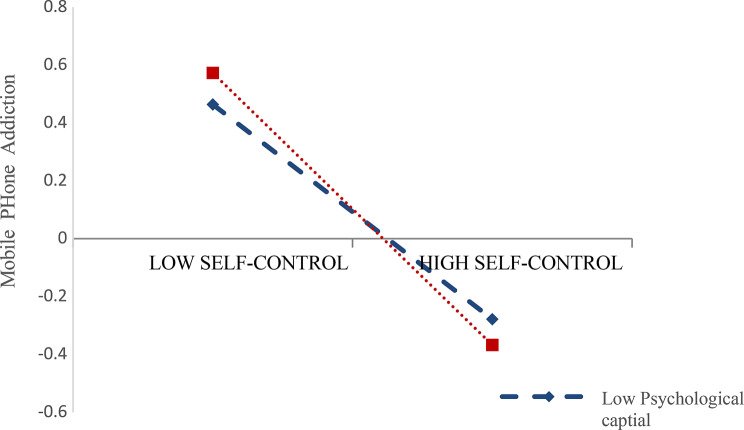
The moderating effect of psychological capital on self-control and mobile phone addiction

Finally, in order to reveal how psychological capital moderates the influence of self-control on mobile phone addiction tendency, a simple slope test was conducted and a simple effect analysis diagram was drawn (Fig. 2) according to the value of psychological capital, which was divided into high and low levels (plus or minus one standard deviation. The results showed that when PPQ score level was low (M-1SD), SCS score could significantly negatively predict MPATS score (*β* = -0.287, *t* = -5.553, *p* < 0.001), and 95% confidence interval was [-0.388,-0.185]. When the PPQ score level was high (M + 1SD), the negative prediction effect of SCS score on MPATS score was still significant but weakened (*β* = -0.469, *t* = -9.621, *p* < 0.001), and the 95% confidence interval was [-0.565,-0.373], as shown in Table 4 for specific results. This result shows that with the improvement of psychological capital level, the influence of self-control on mobile phone addiction tendency increases.

**Table 4:** Analysis of the moderating effect of psychological capital

	Psychological capital	Effect	BootSE	BootLLCI	BootULCI
Indirect effect	M-1SD	-0.287	0.052	-0.388	-0.185
	M	-0.363	0.046	-0.453	-0.273
	M + 1SD	-0.469	0.049	-0.565	-0.373

## Discussion

This study explores the influence of perceived stress on mobile phone addiction, the mediating role of self-control, and the moderating role of psychological capital on the mediating process. The results show that stress perception not only directly affects the tendency of mobile phone addiction but also indirectly affects it through self-control. Psychological capital regulates the latter half of the mediating model. Importantly, this study focuses on nursing undergraduates, a population uniquely exposed to high academic, clinical, and emotional demands, which may exacerbate their vulnerability to mobile phone addiction.

### Analysis of the influence of stress perception on mobile phone addiction tendency of nursing undergraduates

The results of this study show that stress perception is a risk factor for mobile phone addiction tendency in nursing undergraduates. In other words, students who reported more subjective stress were more likely to develop phone addiction than those who reported less or no stress. This finding not only validates hypothesis 1 of this study but is also consistent with previous findings on college students [34]. However, nursing undergraduates face unique stressors, such as the need to balance rigorous academic demands with clinical training and the emotional labor associated with patient care, which may intensify their reliance on mobile phones as a coping mechanism [16, 35]. This study found that stress perception was positively correlated with nursing students’ mobile phone addiction, which verified the “general stress” theory, that is, stress can cause problem behaviors [36]. The higher the degree of stress perception, the easier it is for students to reduce stress through problem behaviors (such as indulging in games). This suggests that stress perception is an influential factor in mobile phone addiction, and nursing educators and managers need to take effective intervention measures to reduce or relieve students’ subjective pressure, so as to reduce the occurrence and development of mobile phone addiction.

### Analysis of the mediating effect of self-control on stress perception and mobile phone addiction tendency

This study found a mediating effect of self-control between perceived stress and mobile phone addiction, confirming hypothesis 2 of this study. This indicates that the higher the stress perception level of nursing undergraduates, the lower their self-control ability, and the more likely they are to have mobile phone addiction. To delve deeper into the mechanisms, we propose that stress perception depletes self-control resources through two primary pathways. First, stress triggers negative emotions such as anxiety and frustration, which require significant cognitive effort to regulate, thereby depleting self-control resources [37–39]. Second, stress induces maladaptive cognitive processes, such as rumination, which further consume self-control resources and impair the ability to resist temptations like excessive mobile phone use [40]. Nursing students, in particular, face unique challenges such as long clinical hours, high-stakes exams, and exposure to patient suffering, which can deplete self-control resources more rapidly compared to students in other disciplines [16, 35]. According to the view of use-satisfaction theory [41], for college students, as a mass medium, mobile phones can bring satisfaction and happiness and effectively transfer the negative emotions caused by pressure. If you over-rely on this psychological experience and do not control your own behavior, it is easy to abuse or overuse mobile phones and eventually develop into mobile phone dependence or addiction. Therefore, it also suggests that reducing the tendency of mobile phone addiction can be started from the perspective of reducing stress perception and improving individual self-control ability.

### The regulating effect of psychological capital

This study found that the mediating effect of stress perception on nursing undergraduates’ mobile phone addiction tendency may be mediated by psychological capital through self-control. Compared with individuals with a lower level of psychological capital, the self-control ability of individuals with a higher level of psychological capital has a stronger influence on the tendency of mobile phone addiction. The practical significance of this finding lies in the role of psychological capital as a protective factor. Psychological capital—comprising self-efficacy, hope, resilience, and optimism—enhances an individual’s ability to cope with stress and replenishes depleted self-control resources [21]. For example, self-efficacy enables students to believe in their ability to manage stress, while resilience helps them recover quickly from setbacks, reducing their reliance on mobile phones as a coping mechanism. For nursing students, psychological capital—comprising self-efficacy, hope, resilience, and optimism—may be particularly critical in mitigating the effects of stress, given the high demands of their training and future profession [16, 35]. It is speculated that respondents’ high psychological capital, such as confidence in personal abilities and performance, willingness to succeed or achieve set goals, ability to bounce back from difficult times, and optimism about the future, mitigated problem behaviors induced by low self-control, such as mobile phone addiction. The emergence of addictive behaviors stems from the weak self-control and self-regulation ability of individuals, and it is often difficult for individuals to restrain their desire for addictive behaviors, which leads to the emergence of addictive behaviors [40]. For nursing undergraduates, stressful events are unavoidable in daily life and study, and individual self-control resources need to be consumed to complete tasks. However, self-control resources are limited within a certain period. When self-control resources are exhausted, the performance of subsequent tasks will decline, leading to self-control failure. As a cognitive resource that can actively predict mental health, psychological capital can be used as a supplement to self-control resources [42]. According to the theory of “self-control resources” [37], huge energy resources can better promote the level of self-control and enable individuals to better exercise self-control ability. Therefore, regulating self-control to improve mobile phone addiction tendency is conditional; it depends on the individual’s psychological capital. The above results provide some clues for the intervention of mobile phone addiction tendency in nursing undergraduates from the perspective of positive psychology, and decision-makers should conduct targeted intervention according to the differences in psychological capital.

### Implications for intervention and future research

Based on the findings of this study and the research progress of previous researchers, several practical intervention measures can be proposed to address mobile phone addiction among nursing undergraduates. **First**,** stress management programs should be integrated into nursing education curricula to help students cope with academic and clinical stressors effectively.** For example, mindfulness-based stress reduction (MBSR) programs have been shown to reduce stress and improve self-control among college students [35, 43]. These programs could be tailored to the unique needs of nursing students, incorporating techniques such as guided meditation, breathing exercises, and cognitive-behavioral strategies to manage stress and prevent maladaptive coping behaviors like excessive mobile phone use.

**Second**,** interventions aimed at enhancing self-control should be prioritized.** Training programs that focus on improving self-regulation skills, such as time management, goal setting, and impulse control, could help nursing students resist the urge to overuse mobile phones [44]. For instance, workshops on self-control strategies, combined with regular self-monitoring of mobile phone usage, could empower students to develop healthier habits [40].Additionally, incorporating digital detox challenges or screen-time tracking apps into nursing programs could encourage students to reflect on their mobile phone usage and set realistic limits.

**Third**,** fostering psychological capital should be a key component of interventions for nursing students.** Given the protective role of psychological capital in mitigating the effects of stress and low self-control, programs that build self-efficacy, hope, resilience, and optimism should be implemented. For example, resilience training workshops and peer support groups could help students develop a positive mindset and strengthen their ability to bounce back from setbacks [16, 45].Encouraging students to engage in activities that promote psychological well-being, such as physical exercise, creative hobbies, and social interactions, could also reduce their reliance on mobile phones for emotional regulation.

**Finally**,** future research should explore the effectiveness of these interventions in reducing mobile phone addiction among nursing students.** Longitudinal studies could examine the long-term impact of stress management, self-control training, and psychological capital enhancement programs on mobile phone usage and academic performance. Additionally, qualitative research could provide deeper insights into the unique stressors faced by nursing students and their coping mechanisms, informing the development of more targeted interventions.

### Theoretical contributions

The findings of this study contribute to several theoretical frameworks. First, they support the General Strain Theory [8], which posits that stress leads to problem behaviors as a coping mechanism. Our results extend this theory by demonstrating that self-control acts as a critical mediator in this process, particularly in the context of mobile phone addiction. Second, the study aligns with the Strength Model of Self-Control [9], which suggests that self-control is a limited resource that can be depleted by stress. Our findings highlight the role of psychological capital as a buffer, providing a novel perspective on how psychological resources can mitigate the negative effects of stress on self-control.

### Limitations and generalizability

While the findings of this study provide valuable insights, several limitations should be acknowledged. First, the cross-sectional design limits our ability to establish causal relationships. Future research should adopt longitudinal or experimental designs to better understand the causal mechanisms underlying these relationships. Second, the, study focused exclusively on nursing undergraduates in China, which may limit the generalizability of the findings to other populations or cultural contexts. For example, cultural differences in mobile phone use patterns and stress levels across educational systems may influence the applicability of these findings. Future studies should explore these relationships in diverse populations to assess their generalizability.

## Conclusion

This study found that self-control and psychological capital had a protective effect on nursing undergraduates’ mobile phone addiction tendency, while stress perception had a negative effect on nursing undergraduates’ mobile phone addiction tendency. This study expanded the influence mechanism of stress perception on mobile phone addiction tendency, explored the influence path of self-control and psychological capital on the relationship between stress perception and mobile phone addiction tendency, and further confirmed the complex interaction between variables. In addition, this study found that for nursing undergraduates with high stress perception, improving self-control ability and psychological capital level is an effective way to improve mobile phone addiction. This study has important theoretical significance for preventing and reducing students’ mobile phone addiction tendency behavior and promoting their healthy development.

## Supplementary Information

Below is the link to the electronic supplementary material.

Supplementary Material 1

Supplementary Material 2

## Data Availability

The datasets generated during and/or analyzed during the current study are available from the corresponding author on reasonable request.

## Abbreviations

PSS: Stress Perception Scale
SCS: Self-control Scale
PPQ: Positive Psychological Capital Questionnaire
MPATS: Mobile Phone Addiction Tendency Scale for College students
