# Supplementary material for: The influence of stress perception on mobile phone addiction tendency in nursing undergraduates: the mediating role of self-control and the moderating role of psychological capital
Source: BMC Nurs. 2025 Aug 25;24:1116. doi: 10.1186/s12912-025-03753-y (PMC12379332; doi:10.1186/s12912-025-03753-y)
Supplement: Supplementary file 1 — Supplementary Material 1 [file 12912_2025_3753_MOESM1_ESM.docx]

**Survey on "The Influence of Perceived Stress on Smartphone Addiction Tendency among Nursing Undergraduates" at a University in Shaanxi Province**

Dear Student,

Hello! This is an anonymous questionnaire for academic research, aiming to understand the situation regarding "The influence of perceived stress on smartphone addiction tendency among nursing undergraduates" at our university. This survey is specifically conducted for this research. The data collected will be used solely for academic purposes and will not adversely affect participants' health.

Participation in this survey is voluntary. If you are willing to participate, please fill it out truthfully based on your actual situation! Thank you for your cooperation!

**Basic Information**

1. Your Gender： [Single Choice]

| ○A. Male | ○B. Female |
| --- | --- |

2. Your Grade: [Single Choice]

| ○A. Freshman | ○B. Sophomore | ○C. Junior | ○D. Senior |
| --- | --- | --- | --- |

**College Students' Mobile Phone Addiction Tendency Scale**

1. If I haven't carried my phone for a while, I will immediately check if there are any text messages/missed calls. [Single Choice]

○ A. Strongly disagree ○ B. Disagree ○ C. Neutral ○ D. Agree ○ E. Strongly agree

2. I prefer chatting on my phone to direct face-to-face communication. [Single Choice]

○ A. Strongly disagree ○ B. Disagree ○ C. Neutral ○ D. Agree ○ E. Strongly agree

3. While waiting for someone, I frequently call them to ask where they are; if I don't call, I feel anxious and restless. [Single Choice]

○ A. Strongly disagree ○ B. Disagree ○ C. Neutral ○ D. Agree ○ E. Strongly agree

4. I feel uncomfortable if I haven't used my phone for a long time. [Single Choice]

○ A. Strongly disagree ○ B. Disagree ○ C. Neutral ○ D. Agree ○ E. Strongly agree

5. In class, I cannot concentrate on listening due to phone calls or text messages. [Single Choice]

○ A. Strongly disagree ○ B. Disagree ○ C. Neutral ○ D. Agree ○ E. Strongly agree

6. I would feel lonely without my phone. [Single Choice]

○ A. Strongly disagree ○ B. Disagree ○ C. Neutral ○ D. Agree ○ E. Strongly agree

7. I feel more confident when communicating with others via mobile phone. [Single Choice]

○ A. Strongly disagree ○ B. Disagree ○ C. Neutral ○ D. Agree ○ E. Strongly agree

8. When my phone doesn't ring for a while, I feel uncomfortable and subconsciously check if there are any missed calls/text messages. [Single Choice]

○ A. Strongly disagree ○ B. Disagree ○ C. Neutral ○ D. Agree ○ E. Strongly agree

9. I often have the illusion that "my phone is ringing/my phone is vibrating". [Single Choice]

○ A. Strongly disagree ○ B. Disagree ○ C. Neutral ○ D. Agree ○ E. Strongly agree

10. Having many calls and text messages makes me feel like my life is fuller. [Single Choice]

○ A. Strongly disagree ○ B. Disagree ○ C. Neutral ○ D. Agree ○ E. Strongly agree

11. I often fear that my phone will automatically shut down. [Single Choice]

○ A. Strongly disagree ○ B. Disagree ○ C. Neutral ○ D. Agree ○ E. Strongly agree

12. My phone is an integral part of me; if I use it less, I feel like something is missing. [Single Choice]

○ A. Strongly disagree ○ B. Disagree ○ C. Neutral ○ D. Agree ○ E. Strongly agree

13. My classmates/friends often say that I rely too much on my phone. [Single Choice]

○ A. Strongly disagree ○ B. Disagree ○ C. Neutral ○ D. Agree ○ E. Strongly agree

14. When my phone frequently has no signal or can't connect, I become anxious and irritable. [Single Choice]

○ A. Strongly disagree ○ B. Disagree ○ C. Neutral ○ D. Agree ○ E. Strongly agree

15. In class, I often voluntarily focus my attention on my phone, which affects my listening. [Single Choice]

○ A. Strongly disagree ○ B. Disagree ○ C. Neutral ○ D. Agree ○ E. Strongly agree

16. I feel more comfortable communicating with others using my phone. [Single Choice]

○ A. Strongly disagree ○ B. Disagree ○ C. Neutral ○ D. Agree ○ E. Strongly agree

**Chinese version of the Perceived Stress Scale**

Please indicate how often you have felt or thought a certain way during the last month.

1. Upset because of something that happened unexpectedly. [Single Choice]

○A. Never ○ B. Occasionally ○ C. Sometimes ○ D. Often ○ E. Always

2. Feeling unable to control important things in your lives. [Single Choice]

○A. Never ○ B. Occasionally ○ C. Sometimes ○ D. Often ○ E. Always

3. Feeling nervous, uneasy, and stressed. [Single Choice]

○A. Never ○ B. Occasionally ○ C. Sometimes ○ D. Often ○ E. Always

4. Successfully dealing with annoying troubles in life. [Single Choice]

○A. Never ○ B. Occasionally ○ C. Sometimes ○ D. Often ○ E. Always

5. Feeling able to effectively handle important changes that occur in life. [Single Choice]

○A. Never ○ B. Occasionally ○ C. Sometimes ○ D. Often ○ E. Always

6. Have confidence in the ability to handle personal issues. [Single Choice]

○A. Never ○ B. Occasionally ○ C. Sometimes ○ D. Often ○ E. Always

7. Feel that things were going your way. [Single Choice]

○A. Never ○ B. Occasionally ○ C. Sometimes ○ D. Often ○ E. Always

8. Found that you could not cope with all the things you had to do. [Single Choice]

○A. Never ○ B. Occasionally ○ C. Sometimes ○ D. Often ○ E. Always

9. There are ways to control annoying things in life. [Single Choice]

○A. Never ○ B. Occasionally ○ C. Sometimes ○ D. Often ○ E. Always

10. Felt that you were on top of things. [Single Choice]

○A. Never ○ B. Occasionally ○ C. Sometimes ○ D. Often ○ E. Always

11. Been angered because of things that happened that were outside of your control.[Single Choice]

○A. Never ○ B. Occasionally ○ C. Sometimes ○ D. Often ○ E. Always

12. Found yourself thinking about things you have to accomplish. [Single Choice]

○A. Never ○ B. Occasionally ○ C. Sometimes ○ D. Often ○ E. Always

13. Been able to control the way you spend your time. [Single Choice]

○A. Never ○ B. Occasionally ○ C. Sometimes ○ D. Often ○ E. Always

14. Frequently feel overwhelmed by mountains of difficulties that seem insurmountable. [Single Choice]

○A. Never ○ B. Occasionally ○ C. Sometimes ○ D. Often ○ E. Always

**Psychological Capital Scale**

1. Many people appreciate my abilities. [Single Choice]

○ A. Strongly disagree ○ B. Disagree ○ C. Somewhat disagree

○ D. Somewhat agree ○ E. Agree ○ F. Strongly agree

2. I don't like to get angry. [Single Choice]

○ A. Strongly disagree ○ B. Disagree ○ C. Somewhat disagree

○ D. Somewhat agree ○ E. Agree ○ F. Strongly agree

3. My insights and abilities exceed those of ordinary people. [Single Choice]

○ A. Strongly disagree ○ B. Disagree ○ C. Somewhat disagree

○ D. Somewhat agree ○ E. Agree ○ F. Strongly agree

4. When encountering setbacks, I can quickly recover. [Single Choice]

○ A. Strongly disagree ○ B. Disagree ○ C. Somewhat disagree

○ D. Somewhat agree ○ E. Agree ○ F. Strongly agree

5. I have great confidence in my abilities. [Single Choice]

○ A. Strongly disagree ○ B. Disagree ○ C. Somewhat disagree

○ D. Somewhat agree ○ E. Agree ○ F. Strongly agree

6. I rarely care about the unpleasantness in life. [Single Choice]

○ A. Strongly disagree ○ B. Disagree ○ C. Somewhat disagree

○ D. Somewhat agree ○ E. Agree ○ F. Strongly agree

7. I always excel in completing tasks. [Single Choice]

○ A. Strongly disagree ○ B. Disagree ○ C. Somewhat disagree

○ D. Somewhat agree ○ E. Agree ○ F. Strongly agree

8. Bad experiences can make me depressed for a long time. [Single Choice]

○ A. Strongly disagree ○ B. Disagree ○ C. Somewhat disagree

○ D. Somewhat agree ○ E. Agree ○ F. Strongly agree

9. When facing difficulties, I will calmly seek solutions. [Single Choice]

○ A. Strongly disagree ○ B. Disagree ○ C. Somewhat disagree

○ D. Somewhat agree ○ E. Agree ○ F. Strongly agree

10. I ofen feel overwhelmed by life stresses. [Single Choice]

○ A. Strongly disagree ○ B. Disagree ○ C. Somewhat disagree

○ D. Somewhat agree ○ E. Agree ○ F. Strongly agree

11. I am willing to take on difficult and challenging work. [Single Choice]

○ A. Strongly disagree ○ B. Disagree ○ C. Somewhat disagree

○ D. Somewhat agree ○ E. Agree ○ F. Strongly agree

12. When things don't go as planned, I tend to feel discouraged. [Single Choice]

○ A. Strongly disagree ○ B. Disagree ○ C. Somewhat disagree

○ D. Somewhat agree ○ E. Agree ○ F. Strongly agree

13. When facing adversity, I will actively try different strategies. [Single Choice]

○ A. Strongly disagree ○ B. Disagree ○ C. Somewhat disagree

○ D. Somewhat agree ○ E. Agree ○ F. Strongly agree

14. When I'm under a lot of pressure, I can't eat well or sleep well. [Single Choice]

○ A. Strongly disagree ○ B. Disagree ○ C. Somewhat disagree

○ D. Somewhat agree ○ E. Agree ○ F. Strongly agree

15. I actively study and work to achieve my ideals. [Single Choice]

○ A. Strongly disagree ○ B. Disagree ○ C. Somewhat disagree

○ D. Somewhat agree ○ E. Agree ○ F. Strongly agree

16. When the situation is uncertain, I always expect good results. [Single Choice]

○ A. Strongly disagree ○ B. Disagree ○ C. Somewhat disagree

○ D. Somewhat agree ○ E. Agree ○ F. Strongly agree

17. I am working hard to achieve my goals. [Single Choice]

○ A. Strongly disagree ○ B. Disagree ○ C. Somewhat disagree

○ D. Somewhat agree ○ E. Agree ○ F. Strongly agree

18. I always see the good side of things. [Single Choice]

○ A. Strongly disagree ○ B. Disagree ○ C. Somewhat disagree

○ D. Somewhat agree ○ E. Agree ○ F. Strongly agree

19. I pursue my goals with confidence. [Single Choice]

○ A. Strongly disagree ○ B. Disagree ○ C. Somewhat disagree

○ D. Somewhat agree ○ E. Agree ○ F. Strongly agree

20. I think good people still make up the vast majority in society. [Single Choice]

○ A. Strongly disagree ○ B. Disagree ○ C. Somewhat disagree

○ D. Somewhat agree ○ E. Agree ○ F. Strongly agree

21. I have a certain plan for my studies and life. [Single Choice]

○ A. Strongly disagree ○ B. Disagree ○ C. Somewhat disagree

○ D. Somewhat agree ○ E. Agree ○ F. Strongly agree

22. Most of the time, I am full of vigor and vitality. [Single Choice]

○ A. Strongly disagree ○ B. Disagree ○ C. Somewhat disagree

○ D. Somewhat agree ○ E. Agree ○ F. Strongly agree

23. I am very clear about what kind of life I want. [Single Choice]

○ A. Strongly disagree ○ B. Disagree ○ C. Somewhat disagree

○ D. Somewhat agree ○ E. Agree ○ F. Strongly agree

24. I think life is beautiful. [Single Choice]

○ A. Strongly disagree ○ B. Disagree ○ C. Somewhat disagree

○ D. Somewhat agree ○ E. Agree ○ F. Strongly agree

25. I don't know what my life goals are either. [Single Choice]

○ A. Strongly disagree ○ B. Disagree ○ C. Somewhat disagree

○ D. Somewhat agree ○ E. Agree ○ F. Strongly agree

26. I think the future is full of hope. [Single Choice]

○ A. Strongly disagree ○ B. Disagree ○ C. Somewhat disagree

○ D. Somewhat agree ○ E. Agree ○ F. Strongly agree

**Self-Control Scale**

1. I am good at resisting temptation. [Single Choice]

○ A. Strongly disagree ○ B. Disagree ○ C. Neutral ○ D. Agree ○ E. Strongly agree

2. I have a hard time breaking bad habits. [Single Choice]

○ A. Strongly disagree ○ B. Disagree ○ C. Neutral ○ D. Agree ○ E. Strongly agree

3. I am lazy. [Single Choice]

○ A. Strongly disagree ○ B. Disagree ○ C. Neutral ○ D. Agree ○ E. Strongly agree

4. I do things that bring me pleasure but are bad for me. [Single Choice]

○ A. Strongly disagree ○ B. Disagree ○ C. Neutral ○ D. Agree ○ E. Strongly agree

5. People can count on me to keep my plans. [Single Choice]

○ A. Strongly disagree ○ B. Disagree ○ C. Neutral ○ D. Agree ○ E. Strongly agree

6. It's hard for me to get up in the morning. [Single Choice]

○ A. Strongly disagree ○ B. Disagree ○ C. Neutral ○ D. Agree ○ E. Strongly agree

7. People say I'm impulsive. [Single Choice]

○ A. Strongly disagree ○ B. Disagree ○ C. Neutral ○ D. Agree ○ E. Strongly agree

8. I spend too much money. [Single Choice]

○ A. Strongly disagree ○ B. Disagree ○ C. Neutral ○ D. Agree ○ E. Strongly agree

9. I get carried away by my feelings. [Single Choice]

○ A. Strongly disagree ○ B. Disagree ○ C. Neutral ○ D. Agree ○ E. Strongly agree

10. I do many things on impluse. [Single Choice]

○ A. Strongly disagree ○ B. Disagree ○ C. Neutral ○ D. Agree ○ E. Strongly agree

11. People say I have iron self-discipline. [Single Choice]

○ A. Strongly disagree ○ B. Disagree ○ C. Neutral ○ D. Agree ○ E. Strongly agree

12. Sometimes fun things distract me and I can't finish tasks on time. [Single Choice]

○ A. Strongly disagree ○ B. Disagree ○ C. Neutral ○ D. Agree ○ E. Strongly agree

13. I have trouble concentrating. [Single Choice]

○ A. Strongly disagree ○ B. Disagree ○ C. Neutral ○ D. Agree ○ E. Strongly agree

14. I can work effectively towards long-term goals. [Single Choice]

○ A. Strongly disagree ○ B. Disagree ○ C. Neutral ○ D. Agree ○ E. Strongly agree

15. Sometimes I can't stop myself from doing things I know are wrong. [Single Choice]

○ A. Strongly disagree ○ B. Disagree ○ C. Neutral ○ D. Agree ○ E. Strongly agree

16. I often act without thinking things through. [Single Choice]

○ A. Strongly disagree ○ B. Disagree ○ C. Neutral ○ D. Agree ○ E. Strongly agree

17. I tend to get angry too easily. [Single Choice]

○ A. Strongly disagree ○ B. Disagree ○ C. Neutral ○ D. Agree ○ E. Strongly agree

18. I often disturb others. [Single Choice]

○ A. Strongly disagree ○ B. Disagree ○ C. Neutral ○ D. Agree ○ E. Strongly agree

19. I sometimes drink excessively (or spend too much time online) . [Single Choice]

○ A. Strongly disagree ○ B. Disagree ○ C. Neutral ○ D. Agree ○ E. Strongly agree
